# Novel surgical technique for complete traumatic rupture of the pancreas: A case report

**DOI:** 10.1186/1752-1947-5-456

**Published:** 2011-09-14

**Authors:** Martin E Kreis, Markus Albertsmeier, Anno Graser, Detlef Krenz, Karl-Walter Jauch, Wolfgang E Thasler

**Affiliations:** 1 Department of Surgery, Ludwig-Maximilian's University, Grosshadern Hospital, Marchioninistraße 15, D-81377 Munich, Germany; 2 Department of Clinical Radiology, Ludwig-Maximilian's University, Grosshadern Hospital, Marchioninistraße 15, D-81377 Munich, Germany; 3 Department of Surgery, Dritter Orden Hospital, Menzinger Straße 44, D-80638 Munich, Germany

## Abstract

**Introduction:**

Complete pancreatic rupture is a rare injury. The typical mechanism by which this occurs is overstretching of the pancreas across the vertebral column during blunt abdominal trauma. The management of this injury depends on the location and extent of the injury.

**Case presentation:**

A 45-year-old Caucasian woman presented with blunt abdominal trauma after she fell onto the end of a handlebar during a bicycle accident. She arrived in the emergency room with stable vital signs and an isolated bruise just above the umbilicus. A computed tomography scan revealed a complete rupture of the pancreas, just ventral to her superior mesenteric vein, and an accompanying hematoma but no additional injuries. An emergency laparotomy was performed; the head of the pancreas was oversewn with interrupted sutures and this was followed by a two-layer pancreaticojejunostomy with the tail of the pancreas. The recovery after surgery was completely uneventful.

**Conclusions:**

Isolated complete pancreatic rupture is a rare injury that can be managed with complete organ preservation. The combination of suturing the pancreatic head and two-layer pancreaticojejunostomy with the pancreatic tail is a feasible technique to manage this condition.

## Introduction

Blunt abdominal trauma is typically followed by laceration of the spleen, liver, or mesentery of the intestine. Such trauma may also cause pancreatic rupture, although this happens in less than 1% of cases [1]. Isolated pancreatic rupture after blunt abdominal trauma is even rarer, and very few case reports (for example, [2]) have been published. Pancreatic rupture may be classified by the Lucas classification [3] from grade I to III (I: superficial contusion with minimal damage; II: deep laceration or transection of the left portion of the pancreas; III: injury of the pancreatic head). Management of pancreatic rupture is controversial. A key question is whether the pancreatic duct was left intact or not. In general, treatment with external drainage is recommended when the duct is intact, whereas distal pancreatectomy is typically suggested for lesions to the main duct in the pancreatic body or tail [4]. Complete rupture of the pancreas is most easily and safely managed by oversewing the proximal part of the pancreas and removing the distal portion (that is, the pancreatic tail) [5]. Distal pancreatectomy, however, involves the risk of subsequent endocrine dysfunction (that is, reduced glucose tolerance or diabetes in up to 50% of patients) [6]. An alternative that allows organ preservation is to drain the pancreatic tail into the stomach [7]. Here, we present a case of a middle-aged woman with an isolated complete pancreatic rupture that was managed successfully by a special surgical anastomotic technique that has not been reported for the treatment of pancreatic rupture.

## Case presentation

**Figure 1:**
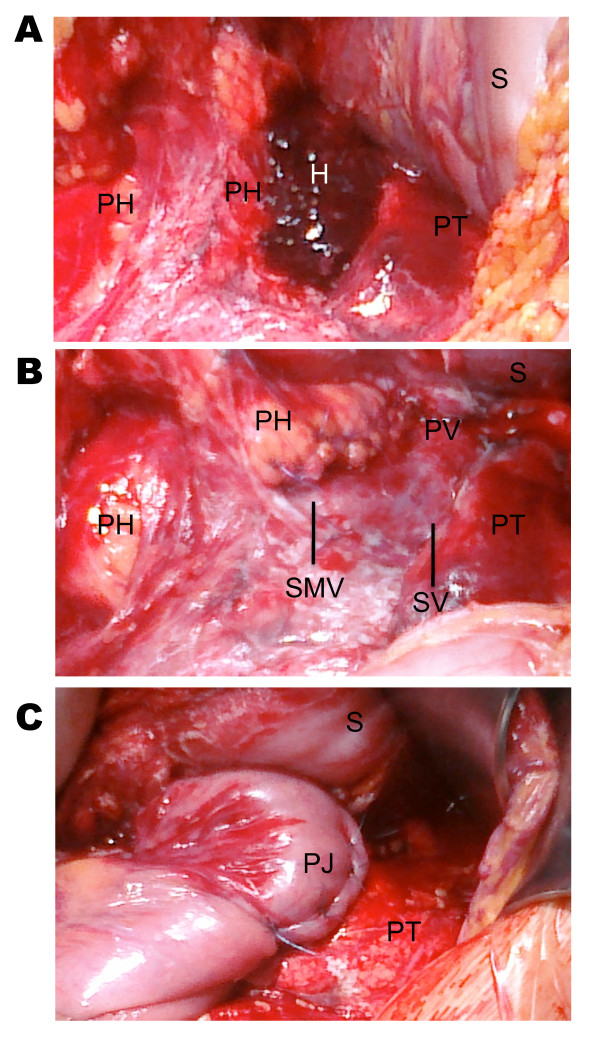
**(A) Intraoperative view after opening of the lesser sac with the stomach (S) lifted upwards**. Note the complete rupture of the pancreas (PH: head of the pancreas; PT: tail of the pancreas). The hematoma (H) accompanying the injury is still in place. (B) The pancreatic duct was closed by a stitch (4-0 Maxon), and the pancreatic head (PH) was oversewn next to the superior mesenteric vein (SMV), which lay open after the hematoma was removed. PT: pancreatic tail; PV: portal vein; S: stomach; SV: splenic vein. (C) This photograph was taken after the tail of the pancreas (PT) was anastomosed as pancreaticojejunostomy (PJ) (two layers) with a Roux-en-Y isolated jejunal loop that was brought to the pancreas behind the transverse colon. S: stomach.

A 45-year-old Caucasian woman accidentally collided with a curb while riding a bicycle. As a result, she fell onto the end of the handlebar and received the impact on her abdomen, just above the umbilicus. She was brought in a stable condition (blood pressure of 110/80 mm Hg and heart rate of 84 beats per minute) to a nearby hospital, where a bruise was noted just above the umbilicus with no symptoms or signs of additional injuries. A computed tomography (CT) scan showed a complete pancreatic rupture ventral to the superior mesenteric vein and accompanying hematoma (Figure 1A). She was referred to our level III trauma center, where an emergency laparotomy was performed. After the lesser sac was opened, a grade IV rupture of the pancreas was confirmed with exposure of the superior mesenteric vein, and no other intra-abdominal injuries were found. The indwelling hematoma was removed, and the open duct at the head of the pancreas was stitched with 4-0 Maxon sutures (Covidien, Dublin, Ireland) and the head was further oversewn with interrupted 4-0 Maxon sutures (Figure 1B). The body and tail of the pancreas were mobilized away from the mesenteric and splenic vein. A bruised slice of pancreas (5 mm) was resected at the tail of the pancreas in order to have vital tissue for the pancreatic anastomosis. A pancreaticojejunostomy was performed with an isolated jejunal loop brought to the pancreas behind the transverse colon by means of the Roux-en-Y technique (Figure 1C) described by Blumgart and colleagues [8]. This two-layer technique consists of an outer full-thickness pancreas-to-seromuscular jejunum anastomosis and an inner duct-to-mucosa anastomosis. The outer layer was prepared by placing U-shaped transpancreatic stitches to the dorsal part of the jejunum while carefully avoiding the pancreatic duct. After the duct-to-mucosa anastomosis was finished by single stitches, the transpancreatic stitches were anchored to the ventral part of the jejunum and this resulted in "sandwiching" of the pancreas within the jejunal wall. Finally, the lesser sac was drained by three 30-French Robinson drains (Smith Medical Deutschland GmbH, Grasbrunn, Germany) before the abdomen was closed.

Consistent with our policy in patients undergoing pancreaticoduodenectomy, octreotide was given for seven days after surgery at a dose of 100 μg three times a day as the pancreas was soft and our patient did not have any contraindications for octreotide treatment. On the day after surgery, the indwelling drains contained high levels of amylase (> 25,000 U/l) and lipase (> 80,000 U/l). The high levels decreased to normal serum levels within four days. The drains were finally removed at day seven after surgery, and the postoperative course was completely uneventful.

## Discussion

Blunt abdominal trauma with isolated rupture of the pancreas is a rare injury [1]. The key criterion for deciding on a treatment option is whether the main pancreatic duct is damaged or not. The value of CT scans, in this regard, is limited by low sensitivity [9]. Although endoscopic retrograde pancreatography or magnetic resonance imaging pancreatography is ideal for diagnosing an injury to the pancreatic duct [9], neither is available on an emergency basis in most hospitals. Early laparotomy appears to be the best option for patients with lesions to the pancreatic duct given that delayed surgical treatment is accompanied by increased morbidity and mortality [10]. Also, late consequences, such as pancreatic cysts, that necessitate further therapy are likely to develop [11]. However, if the pancreatic duct is not involved, mere drainage and a watch-and-wait strategy are usually sufficient [10].

Preoperative imaging of our patient by CT scan revealed complete pancreatic rupture ventral to the superior mesenteric vein. As a result, injury to the main pancreatic duct was obvious and laparotomy was clearly indicated. Different surgical options to manage traumatic pancreatic rupture exist. Injury to the pancreatic head is best managed by drainage and, in some cases, by staged Kausch-Whipple procedure [1,5]. For rupture at the level of the pancreatic body and tail (most frequently seen following blunt abdominal trauma), most surgeons choose to perform a distal pancreatectomy [1], which may be combined with Roux-en-Y pancreaticojejunostomy to reduce the risk of postoperative pancreatic fistula [12]. In our patient, the pancreas was ruptured between the head and body so that distal pancreatectomy would have resulted in a severe loss of pancreatic tissue and a significant risk of subsequent endocrine or exocrine dysfunction or both [6]. However, we would recommend a quick distal pancreatectomy in an unstable patient. As our patient was stable and had an isolated pancreatic rupture, we chose to perform an organ-preserving operation: the proximal portion of the pancreatic head was oversewn by interrupted sutures, and the duct was closed with a separate stitch. An anastomosis to the pancreatic head was unfavorable in this case as the rupture was so proximal that a telescoping anastomosis was not possible. The pancreatic body was mobilized easily and anastomosed into a Roux-en-Y isolated jejunal loop in accordance with the technique described by Blumgart and colleagues [8] for pancreaticojejunostomy during Kausch-Whipple operations. In this technique, transpancreatic sutures are used because they can be anchored to the jejunal wall and not within the pancreas; this is advantageous when the pancreas is soft, as was the case in our patient. Also, she was treated with octreotide, although no clear evidence for its use is available in the treatment of pancreatic injuries [13,14].

## Conclusions

We conclude that distal pancreatectomy and its potential long-term consequences with regard to endocrine and exocrine function may be successfully circumvented in patients with complete rupture at the level of the pancreatic body. One technique to elegantly achieve this goal is a duct-to-mucosa anastomosis and subsequent transpancreatic U-stitches to "sandwich" the pancreas into the jejunum, resulting in a two-layer pancreaticojejunostomy to the distal pancreas.

## Abbreviations

CT: computed tomography.

## Consent

Written informed consent was obtained from the patient for publication of this case report and any accompanying images. A copy of the written consent is available for review by the Editor-in-Chief of this journal.

## Competing interests

The authors declare that they have no competing interests.

## Authors' contributions

MEK wrote the first version of the manuscript. MA documented the intraoperative findings and arranged the details of the figures. All authors reviewed the manuscript, made several contributions to amend and improve the case presentation, and read and approved the final manuscript. All authors reviewed the manuscript, made several contributions to amend and improve the case presentation substantially, and read and approved the final manuscript.
